# Integrated metabolomic and transcriptomic analyses reveal that starch and sucrose metabolism regulate maize kernel hardness

**DOI:** 10.3389/fpls.2025.1688375

**Published:** 2025-11-24

**Authors:** Haiyu Zhou, Xiaodong Xie, Hexia Xie, Lanqiu Qin, Yuxin Xie, Xiang Yang, Bujin Zhou, Jingdan He, Bingwei Wang, Chengqiao Shi, Juzhi Lv, Xianjie Tan, Jinguo Zhou, Weidong Cheng, Yufeng Jiang

**Affiliations:** Maize Research Institute, Guangxi Academy of Agricultural Sciences, Nanning, China

**Keywords:** maize kernel, metabolomic, transcriptomic, carotenoids, differentially expressed genes

## Abstract

Maize kernel hardness, largely determined by the structural and compositional characteristics of the endosperm, is a key trait affecting grain quality, milling performance, and storage stability. Although vitreous and starchy endosperms exhibit markedly different physical properties, the underlying metabolic and transcriptional mechanisms—particularly the crosstalk between primary and specialized metabolic pathways—remain insufficiently understood. In this study, we conducted integrated metabolomic and transcriptomic analyses, along with cytological observations, to investigate these mechanisms using two contrasting maize inbred lines (D003, vitreous; D009, starchy) at three kernel developmental stages (18, 25, and 32 days after pollination, DAP). Cytological examination revealed that D003 endosperms comprise smaller, tightly packed cells containing polygonal starch granules, whereas D009 endosperms consist of larger, irregular cells with loosely arranged spherical starch granules. Metabolomic profiling revealed significantly higher levels of carotenoids—including carotenes (α-carotene, β-carotene) and xanthophylls (zeaxanthin, lutein)—in D003 kernels across all stages, implicating carotenoid biosynthesis in contributing to kernel hardness. Transcriptomic analysis identified starch and sucrose metabolism as the most significantly enriched pathway among differentially expressed genes (DEGs), with qRT-PCR validation confirming the downregulation of a key sucrose synthase gene (*Zm00001eb313170*). We propose a synergistic model in which transcriptional regulation of starch and sucrose metabolism—particularly reduced sucrose synthase activity—promotes the formation of a compact endosperm structure characterized by polygonal starch granules, while enhanced carotenoid accumulation reinforces cellular interfaces, collectively enhancing kernel hardness. These findings offer novel molecular targets for breeding strategies aimed at improving maize kernel quality.

## 1 Introduction

Maize (*Zea mays* L.) is one of the most widely cultivated crops worldwide. Its kernels serve as a major food source and constitute a staple in the diets of millions across Latin America, Asia, and Africa (West, 2002; Ranum et al., 2014). The mature maize kernel consists of an embryo and a much larger endosperm, both encased by the seed coat (Gibbon and Larkins, 2005). Kernel hardness, predominantly governed by the structural and compositional attributes of the endosperm, is a crucial trait affecting grain quality, milling efficiency, and storage durability (Fox and Manley, 2009). The endosperm comprises two distinct regions: the vitreous (glassy) and the starchy endosperm (Gibbon and Larkins, 2005; Wang et al., 2020). These regions differ markedly in their physical properties, attributed to variations in protein matrix continuity, starch granule compaction, and cell wall architecture (Gerde et al., 2017; Caballero-Rothar et al., 2019). The vitreous endosperm, characterized by higher light transmittance, elevated protein content, and densely packed starch granules, imparts greater durability and resistance to mechanical damage (Wang et al., 2012). However, the molecular and metabolic bases of endosperm hardness remain incompletely understood, particularly regarding the interactions between core metabolic pathways that regulate this trait, and how their coordinated actions at transcriptional and metabolic levels determine the final kernel hardness.

Carotenoids, lipophilic pigments abundantly present in maize kernels (Yin et al., 2024), serve essential roles as vitamin A precursors, antioxidants, and immune modulators in human nutrition (Eggersdorfer and Wyss, 2018; Watkins and Pogson, 2020). Notably, over 70% of kernel carotenoids accumulate in the vitreous endosperm (Ndolo and Beta, 2013; Lozano-Alejo et al., 2007), with distinct compositional profiles between vitreous (β-branch: zeaxanthin, β-cryptoxanthin, β-carotene) and starchy (α-branch: lutein, α-cryptoxanthin, β-carotene) regions, influencing both kernel flour color and texture (Kljak et al., 2014; Saenz et al., 2020; Li and Van Eck, 2007; Saenz et al., 2021). Genetic studies have linked carotenoid metabolism to kernel hardness regulation; for example, *Ven1* regulates β-carotene accumulation, which stabilizes starch granule membranes and modulates protein–starch interactions (Wang et al., 2020), while allelic variants of *ZmPTOX* impact carotenoid biosynthesis (Yin et al., 2024). Nonetheless, the interactions between carotenoid biosynthetic pathways and primary carbon metabolism in determining kernel hardness remain unexplored.

Starch and sucrose metabolism are central to maize kernel development, providing carbon substrates essential for endosperm filling. Starch biosynthesis involves a coordinated network of enzymes, including ADP-glucose pyrophosphorylase (AGPase), starch synthase (SS), and starch branching enzyme (SBE), all of which influence endosperm structure (Jeon et al., 2010; Pfister and Zeeman, 2016; Huang et al., 2021). Simultaneously, the cleavage of sucrose by invertases and sucrose synthases yields hexoses that not only serve as precursors for starch biosynthesis and specialized metabolites but also act as signaling molecules regulating endosperm development (Yang et al., 2024, 2025). Recent studies have partially elucidated the regulatory network underlying endosperm filling, particularly the role of *Opaque2*, a central transcription factor, in coordinating sugar signaling and abscisic acid (ABA) pathways (Yang et al., 2021, 2022; Dai et al., 2021). Furthermore, the interplay between sugar metabolism and other pathways, such as carotenoid biosynthesis, is emerging as a key area of interest (Villwock et al., 2024; Zhang et al., 2025). Despite this progress, the contributions of dynamic starch and sucrose metabolism to kernel development—especially their role in determining endosperm hardness at both transcriptional and metabolic levels and their potential crosstalk with specialized metabolism like carotenoid biosynthesis—remain unclear.

Advancements in omics technologies now allow for comprehensive analyses of complex plant traits (Lu et al., 2023; Zeng et al., 2024). Metabolomics captures snapshots of metabolic flux, while transcriptomics reveals the gene expression landscapes associated with phenotypic variation (Xiao et al., 2024; Nie et al., 2024). While integrated multi-omics approaches have been successfully applied to unravel regulatory networks in various plant systems, few studies have focused on dissecting the molecular basis of maize kernel hardness using such strategies, particularly by simultaneously profiling transcriptional and metabolic changes during key developmental stages. In this study, we employed combined metabolomic and transcriptomic analyses to investigate how starch and sucrose metabolism, along with carotenoid accumulation, influence kernel hardness in maize. By comparing two inbred lines (D003, vitreous; D009, starchy) across key developmental stages, we identified differentially accumulated metabolites (DAMs) and differentially expressed genes (DEGs) associated with carbohydrate metabolism and carotenoid biosynthesis. The primary objectives of this study were to 1) characterize the cytological differences in endosperm structure between vitreous and starchy kernels; 2) identify key metabolic pathways, particularly starch and sucrose metabolism and carotenoid biosynthesis, associated with kernel hardness through integrated metabolomic and transcriptomic analyses; and 3) validate the expression of candidate genes involved in these pathways to elucidate their potential roles in regulating endosperm texture. Our findings highlight key metabolic pathways and regulatory genes involved in kernel texture, providing valuable insights for the genetic enhancement of maize grain quality through breeding and biotechnology.

## 2 Materials and methods

### 2.1 Plant materials

The maize inbred lines D003 and D009 were cultivated in the field of the Maize Research Institute of Guangxi Academy of Agricultural Sciences (22°61′N, 108°24′E) in 2024. The natural cultivation environment of the field was used for conducting a completely randomized block design with three replications. Each row was 3 m long, and the distance between rows was 0.65 m. The planting density was approximately 71,800 plants per hectare (Zhou et al., 2024). Fresh kernel samples were collected from 18, 25, and 32 days after pollination (DAP) for morphological tests, scanning electron microscopy (SEM), paraffin sections, and physiological and biochemical analyses. At the same time, samples from the same period were rapidly frozen in liquid nitrogen for the next step of metabolite and gene expression analysis.

### 2.2 Scanning electron microscopy

Fresh maize kernels were fixed in 2.5% glutaraldehyde, vacuumed overnight, and stored at 4 °C for 3 days. The samples were then dehydrated in a graded ethanol series: 30%, 50%, 70%, 80%, 90%, and 100%. Next, the samples were soaked in an isopentyl acetate (C_7_H_14_O_2_):ethanol = 1:1 mixture for 10 min and then in pure isopentyl acetate for 10 min. Then, they were dried at 37°C for 5 h, mounted on SEM stubs using conductive tape, sputter-coated with 10–15 nm of gold layer, and imaged using a field-emission SEM (FEI Quattro S) at 5 kV, based on a previously published method (Xu et al., 2024).

### 2.3 Paraffin section

Fresh maize kernels were fixed in 2.5% glutaraldehyde, vacuumed overnight, and stored at room temperature for 3 days. A scalpel was used to smooth the target tissues in the fume hood, and together with the corresponding label, the cut tissues were placed into an embedding frame. The samples were then dehydrated in an ethanol series: 75% alcohol for 4 h, 85% alcohol for 2 h, 90% alcohol for 2 h, 95% alcohol for 1 h, and anhydrous ethanol for 1 h. The samples were also dehydrated in alcohol benzene for 10 min, cleared in xylene for 10 min, and infiltrated in molten paraffin I at 65 °C for 1 h. Next, they were cooled in a −20 °C freezing platform. After the wax had solidified, the wax block was removed from the embedding frame and repaired. The trimmed wax block was placed into a paraffin slicer (LEICA RM2016) for slicing with a thickness of 4 μm and imaged using a microscope (ZEISS Scope.A1).

### 2.4 Targeted metabolomics analysis

Metabolite profiling was conducted through a targeted metabolomic approach by Wuhan Metware Biotechnology Co., Ltd. (Wuhan, China) (http://www.metware.cn/). Freeze-dried kernel samples were crushed using a Mixer Mill (MM 400, Retsch) with a zirconia bead for 1.5 min at 30 Hz. Then, 50 mg of the pulverized kernel powder was weighed and extracted with 0.5 mL of a mixed solution of n-hexane:acetone:ethanol (1:1:1, v/v/v). The extract was vortexed for 20 min at room temperature. Following centrifugation at 12,000 r/min for 5 min at 4°C, the supernatants were collected. The residue was re-extracted under the same conditions. The combined supernatant was evaporated to dryness and reconstituted in 100 µL of dichloromethane. The solution was filtered through a 0.22-µm pore size membrane before being analyzed using an LC-MS/MS system (Melendez-Martinez, 2019; Bartley and Scolnik, 1995; Inbaraj et al., 2008).

Two microliters of the sample was injected into a YMC C30 column (3 µm, 100 mm × 2.0 mm i.d.) operating at 28 °C and a flow rate of 0.8 mL/min. The mobile phases used were methanol:acetonitrile (1:3, v/v) with 0.01% BHT and 0.1% formic acid (phase A) and methyl tert-butyl ether with 0.01% BHT (phase B). The compounds were separated using the following gradient: started at 0% B (0–3 min), increased to 70% B (3–5 min), then increased to 95% B (5–9 min), and finally ramped back to 0% B (10–11 min). An ESI-triple quadrupole-linear ion trap (QTRAP) mass spectrometer (QTRAP^®^ 6500+, AB Sciex) equipped with an APCI heated nebulizer was used, operating in positive ion mode and controlled by Analyst 1.6.3 software (Sciex). The APCI source operation parameters were as follows: source temperature set at 350°C and curtain gas (CUR) set at 25.0 psi. Carotenoids were analyzed using scheduled multiple reaction monitoring (MRM). Data acquisitions were performed using Analyst 1.6.3 software (Sciex). Multiquant 3.0.3 software (Sciex) was used to quantify all metabolites.

The hierarchical cluster analysis (HCA) results of the samples and metabolites were presented as heatmaps with dendrograms. HCA was carried out by the R package pheatmap. For HCA, normalized signal intensities of metabolites (unit variance scaling) were visualized as a color spectrum. Significantly regulated metabolites between groups were determined by absolute Log_2_FC (fold change). Identified metabolites were annotated using the KEGG compound database (http://www.kegg.jp/kegg/compound/), and annotated metabolites were then mapped to the KEGG Pathway database (http://www.kegg.jp/kegg/pathway.html). Pathways with significantly regulated metabolites mapped to them were then fed into MSEA (metabolite sets enrichment analysis), and their significance was determined by the hypergeometric test’s *P*-values.

### 2.5 Transcriptomics analysis

Total RNA was extracted from fresh maize kernels using ethanol precipitation and CTAB-PBIOZOL methods. RNA quality was assessed using a Qubit fluorescence quantifier and a Qsep400 high-throughput biofragment analyzer. The enriched mRNA was fragmented and reverse-transcribed into cDNA using the NEBNext Ultra RNA Library Prep Kit for Illumina. Purified double-stranded cDNA fragments were end-repaired, and a base was added and ligated to Illumina sequencing adaptors. The ligation reaction was purified using AMPure XP Beads. The ligated fragments were subjected to size selection and polymerase chain reaction (PCR). The resulting cDNA library was sequenced on the Illumina platform, yielding 150 bp paired-end reads.

High-quality reads were obtained after filtering the original sequencing data using fastp, and the transcriptome was assembled using StringTie. The reference genome and its annotation files were downloaded from https://download.maizegdb.org/Zm-B73-REFERENCE-NAM-5.0/. HISAT was used to align clean reads to the reference genome. Gene expression levels were quantified using featureCounts, and FPKM values were calculated. DESeq2 was used for differential gene expression analysis between two groups, and the Benjamini–Hochberg correction was applied to *P*-values. Corrected *P*-values and log2 fold change were used as thresholds for significant differential expression. Enrichment analysis was performed based on the hypergeometric test, with pathway-based hypergeometric distribution testing for KEGG and GO term-based analysis for GO. GATK was used for variant site analysis, and ANNOVAR was used for variant annotation. GSEA was conducted using the GSEA tool for gene set enrichment analysis.

### 2.6 Quantitative reverse transcription-polymerase chain reaction analysis

The differential expression of eight structural genes involved in starch and sucrose metabolism from the RNA‐seq data was selected for confirmation by quantitative reverse transcription (qRT)‐PCR. The maize GADPH actin gene was used as a reference to normalize gene expression in all experiments. Total RNA was extracted using the FastPure Universal Plant Total RNA Isolation Kit (Vazyme RC411), and cDNA was synthesized from total RNA using the HiScript III 1st Strand cDNA Synthesis Kit (Vazyme R312). qRT‐PCR amplification was performed according to the ChamQ Universal SYBR qPCR Master Mix (Vazyme Q711). Each amplification system contained 10 μL of ChamQ Universal SYBR qPCR Master Mix, 0.5 μL each of gene‐specific primer (10 μmol L^−1^), and 2 μL of diluted cDNA in a final volume of 20 μL made with sterile-distilled water. The program of each reaction was 95°C for 30 s followed by 40 cycles of 95°C for 10 s and 60°C for 30 s. The relative expression level of each gene was calculated using the 2^−ΔΔCt^ method. Three biological replicates were analyzed for each sample. The primers used for qRT‐PCR are listed in **Supplementary Table S1**.

### 2.7 Statistical analysis and graphing

The data obtained from the experiments were analyzed using Microsoft Excel 2010. The results, presented as the mean ± standard deviation, were visualized using SPSS software version 25 (IBM, Chicago, IL, USA). One-way analysis of variance and Duncan’s multiple-range test were performed to analyze the significance of differences between the groups, and ^**^ in the figures indicates a significant difference (*P* < 0.01). The graphics were illustrated using GraphPad Prism 8 and Adobe Illustrator 2020.

## 3 Results

### 3.1 Maize inbred lines D003 and D009 display differences in endosperm vitreousness

**Figure 1:**
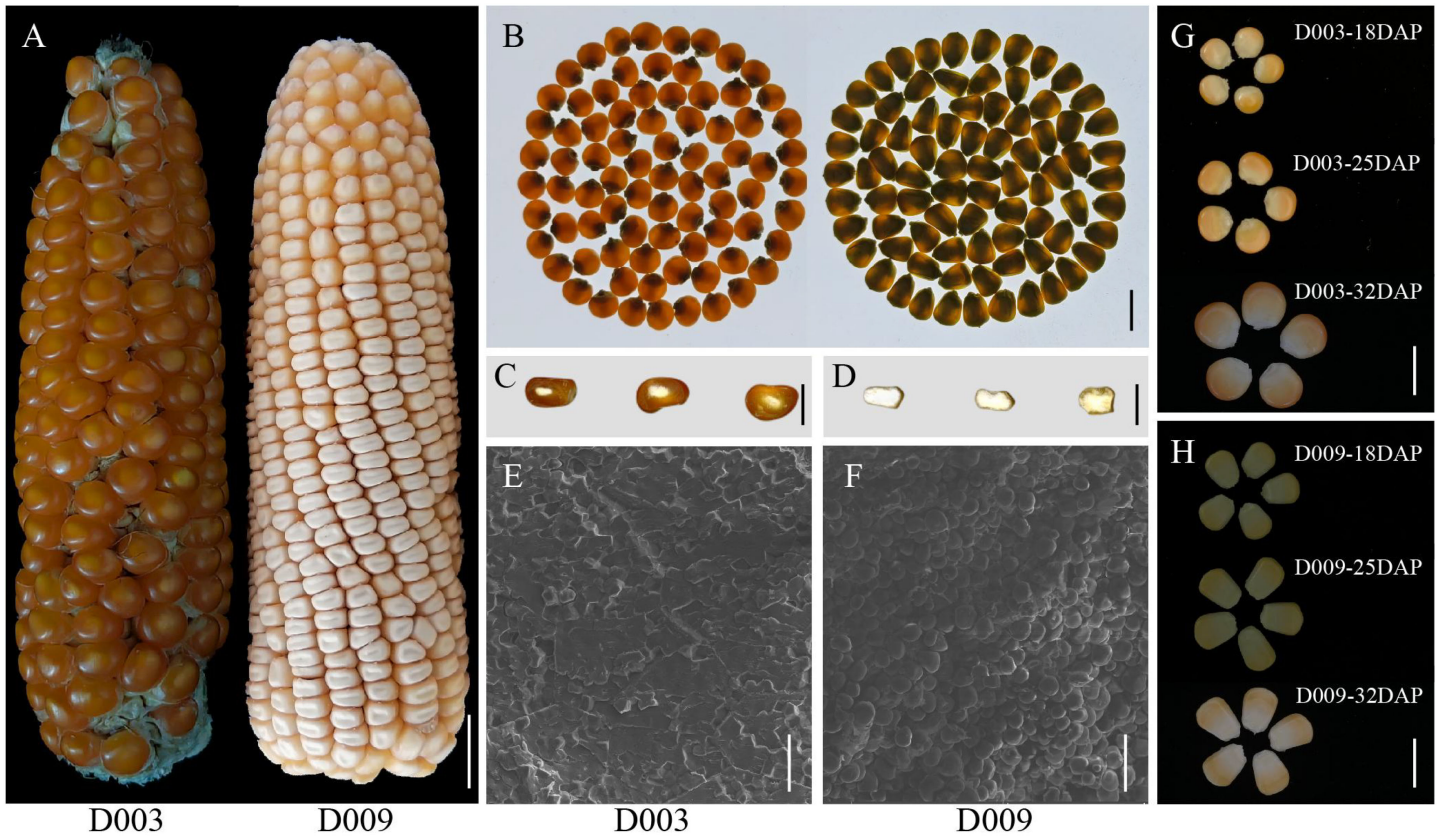
Phenotypic comparison of maize inbred lines D003 and D009. **(A)** Ear phenotypes of D003 and D009. Scale bar, 1 cm. **(B)** Kernel vitreousness as observed on the light box: D003 kernel is vitreous, and D009 kernel is opaque. Scale bar, 1 cm. **(C, D)** Kernel cross sections of D003 and D009. Scale bar, 1 cm. The kernels were cut cross section in the middle with a Chinese herbal knife. **(E)** D003 endosperm starch grain observed by SEM. Scale bar, 20 μm. **(F)** D009 endosperm starch grain observed by SEM. Scale bar, 20 μm. **(G)** D003 kernel morphology at 18, 25, and 32 DAP. Scale bar, 1 cm. **(H)** D009 kernel morphology at 18, 25, and 32 DAP. Scale bar, 1 cm.

To identify the differences in kernel hardness among different maize varieties, we selected the representative inbred lines D003 and D009 in tropical and subtropical maize regions. In the mature period, D003 kernels were red and hard in appearance, while D009 kernels were light yellow and soft (**Figure 1A**). On a light box, light transmission was observed for D003 but not for D009 kernels (**Figure 1B**). The kernel transverse sections show that D003 had an almost entirely vitreous endosperm, while D009 had an almost entirely starchy endosperm (**Figures 1C, D**). Scanning electron microscopy (SEM) revealed that the region of D003 endosperm contained starch granules embedded in a protein matrix, but this matrix was not apparent in D009, which had loosely compacted starch granules (**Figures 1E, F**). These results indicate that D003 and D009 show significant differences in kernel hardness and especially endosperm vitreousness.

### 3.2 Cytological analysis determined that endosperm cells and starch granules affected kernel hardness

**Figure 2:**
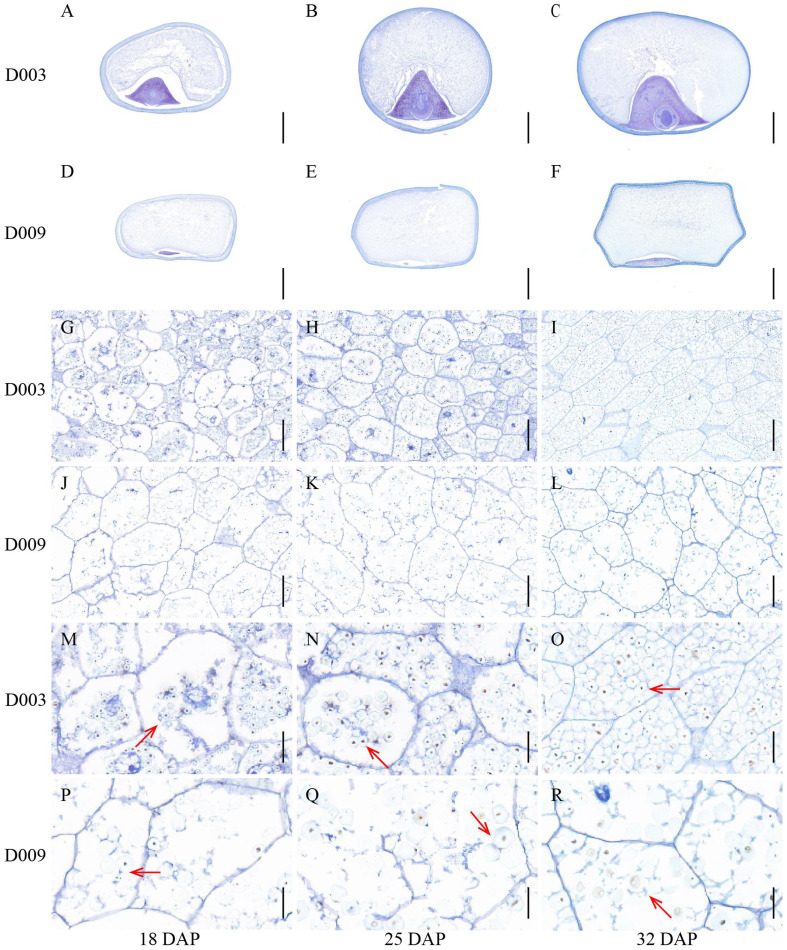
Comparison of D003 and D009 kernel transverse paraffin sections. **(A–C)** D003 kernel transverse section at 18, 25, and 32 DAP. Scale bar, 2 mm. **(D–F)** D009 kernel transverse section at 18, 25, and 32 DAP. Scale bar, 2 mm. **(G–I)** D003 endosperm cells at 18, 25, and 32 DAP. Scale bar, 100 μm. **(J–L)** D009 endosperm cells at 18, 25, and 32 DAP. Scale bar, 100 μm. **(M–O)** D003 endosperm cells were enlarged at 18, 25, and 32 DAP. Scale bar, 30 μm. **(P–R)** D009 endosperm cells were enlarged at 18, 25, and 32 DAP. Scale bar, 30 μm. Note: The red arrows highlight the starch granules.

**Figure 3:**
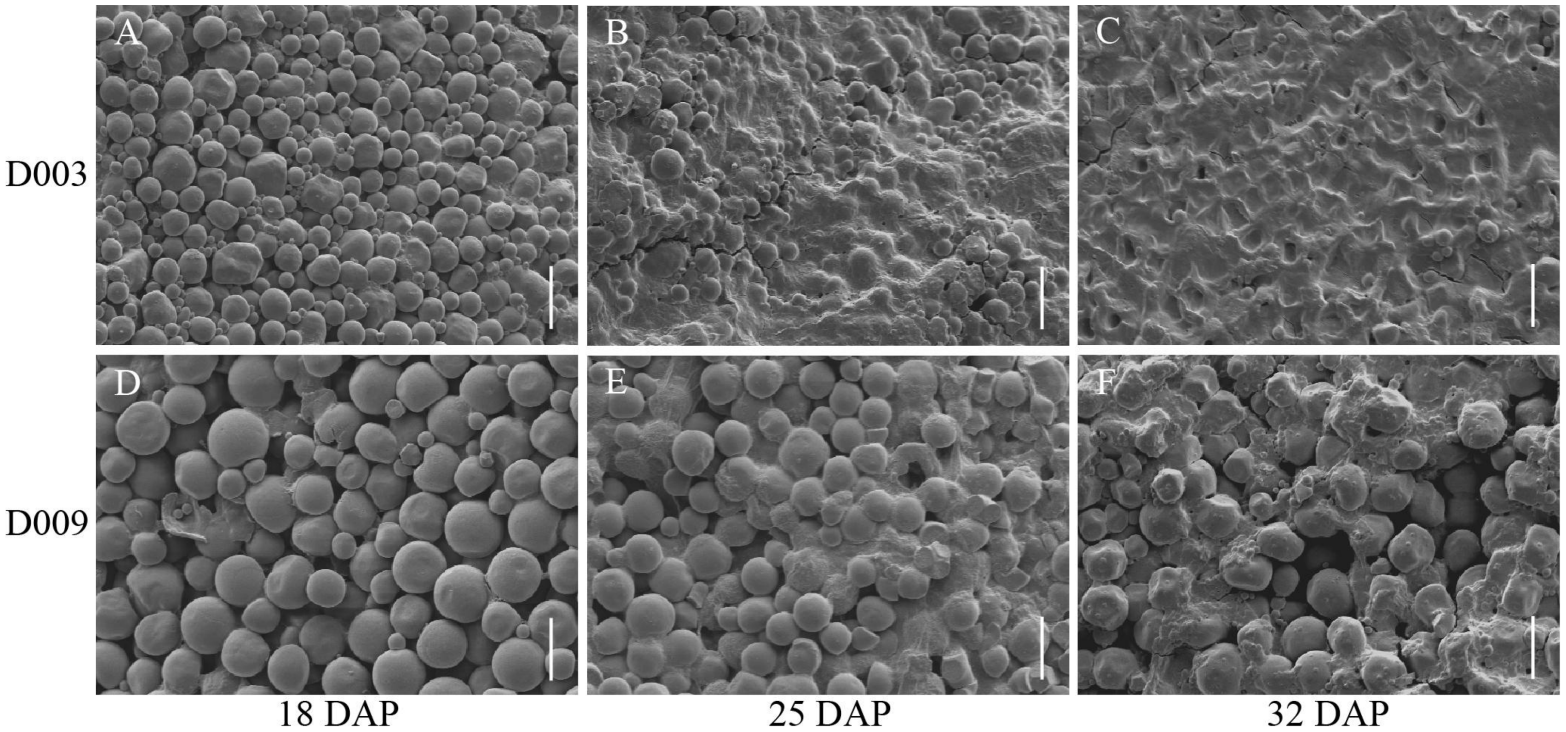
SEM comparison of the endosperm from D003 and D009. **(A–C)** D003 endosperm cell starch granules at 18, 25, and 32 DAP. Scale bar, 20 μm. **(D–F)** D009 endosperm cell starch granules at 18, 25, and 32 DAP. Scale bar, 20 μm.

To investigate the mechanisms of vitreous endosperm formation, we collected kernels at 18, 25, and 32 DAP for further analysis (**Figures 1G, H**, **2A–F**). At 18 DAP, the starch granules inside D003 endosperm cells began to develop (**Figures 2G, M**). At 25 DAP, the starch granules were developing rapidly and increased substantially (**Figures 2H, N**). At 32 DAP, the endosperm cells were tightly packed, and starch granules filled up the whole cells (**Figures 2I, O**). In contrast, the endosperm cells and starch granules in D009 behaved differently. Although D009 began to develop at 18 DAP (**Figures 2J, P**), there was no significant increase in starch grain number at 25 and 32 DAP (**Figures 2K, Q**). At 32 DAP, the endosperm cells were large and irregularly arranged, and the starch granules were loose inside the cells (**Figures 2L, R**). We also observed the ultrastructure of the starch granules by SEM. In D003, as the kernels continuously develop and mature, probably due to tight compacting, the starch granules began to form a polygonal shape (**Figures 3A–C**). However, in D009, the majority of starch granules maintained their spherical morphology (**Figures 3D–F**). These results demonstrated that smaller and closely packed endosperm cells and polygonal shape starch granules tend to facilitate the formation of vitreous endosperm.

### 3.3 Metabolomics analysis confirmed that carotenoids were beneficial for increasing endosperm hardness

**Figure 4:**
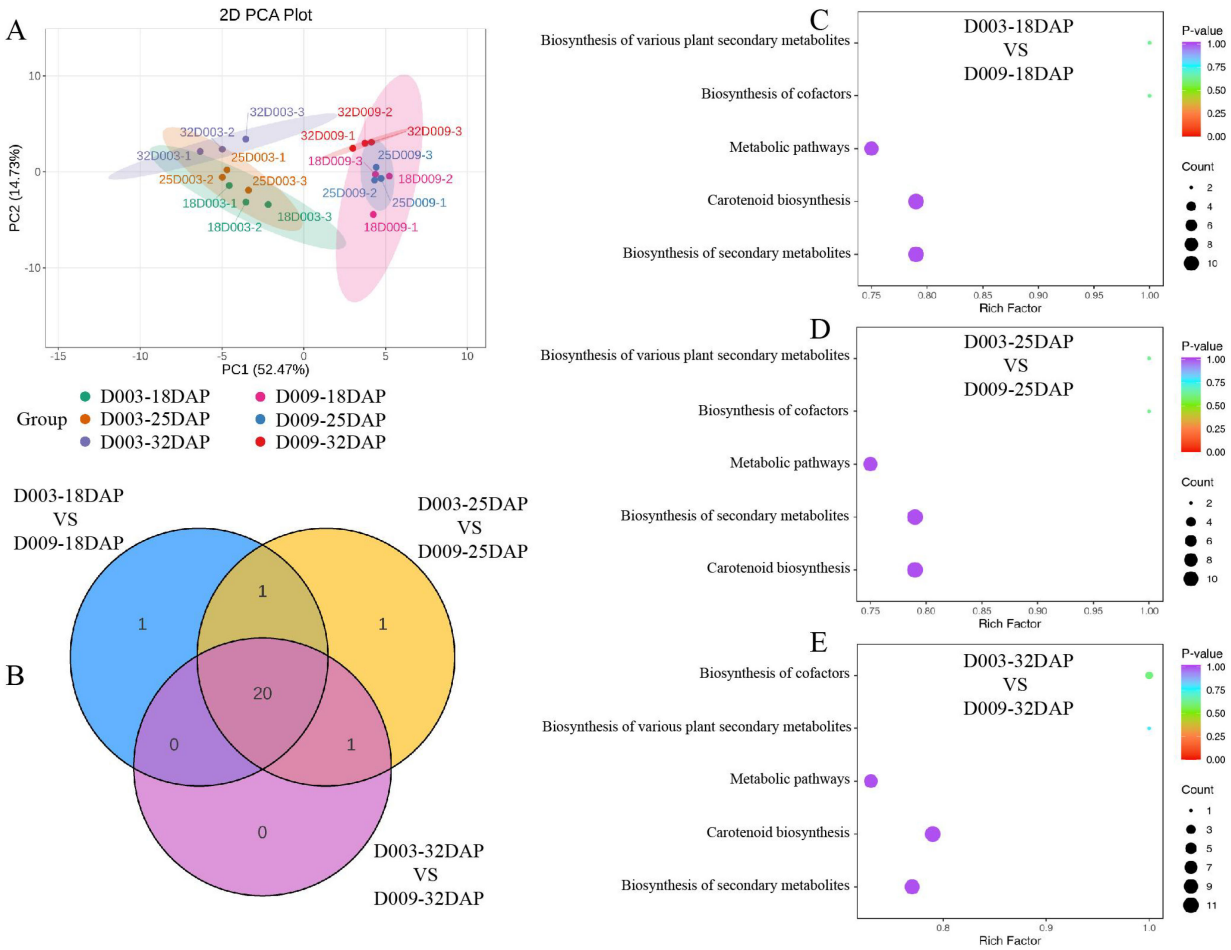
Metabolite profiling of D003 and D009. **(A)** PCA of metabolome data derived from D003 and D009 kernel during three developmental stages (18, 25, and 32 DAP). **(B)** Venn diagram of DAMs. **(C)** KEGG enrichment analysis of DAMs at 18 DAP. **(D)** KEGG enrichment analysis of DAMs at 25 DAP. **(E)** KEGG enrichment analysis of DAMs at 32 DAP. The color of the circle denotes the *P*-value, and the size of the circle denotes the number of DAMs in the pathway.

**Figure 5:**
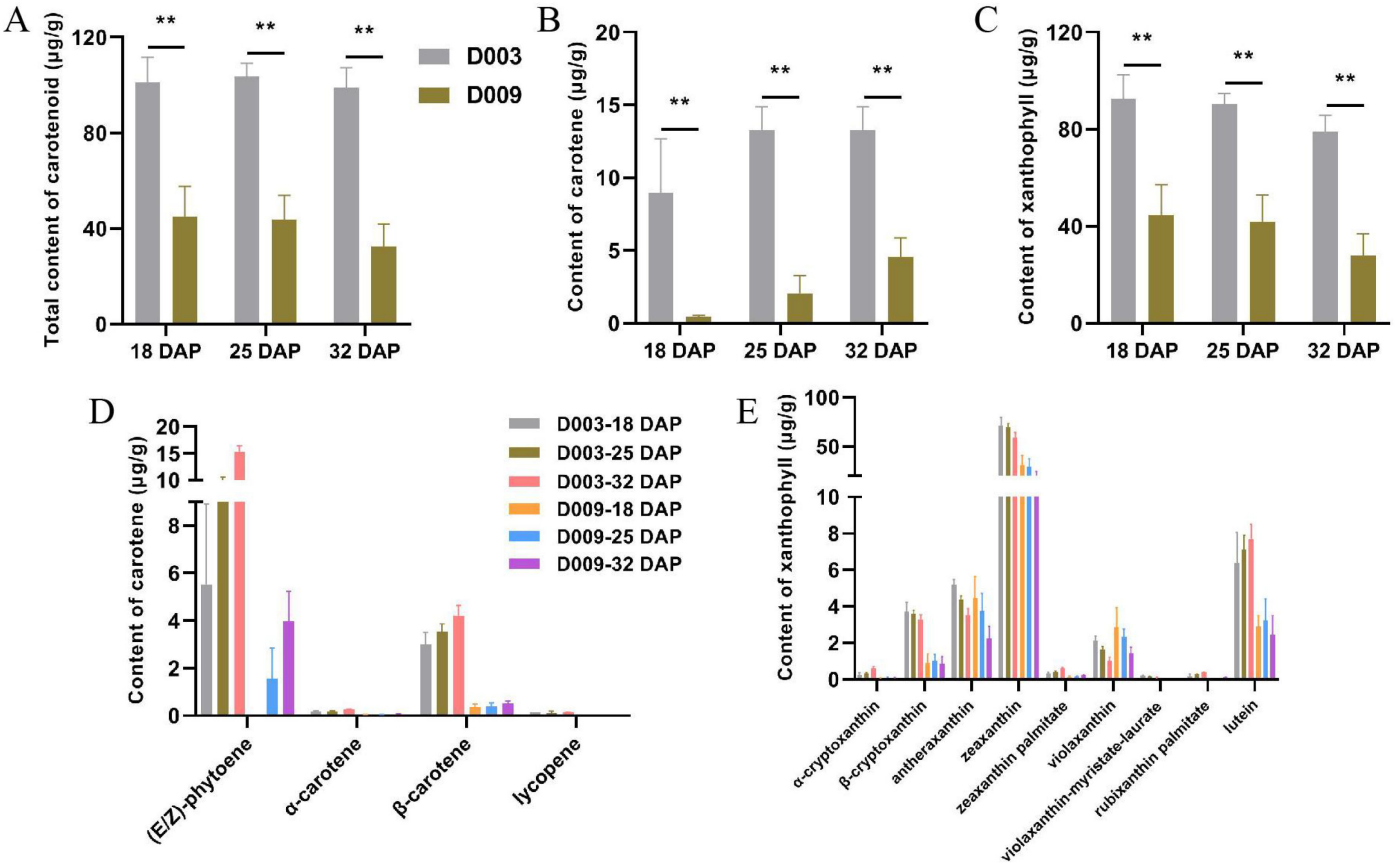
Carotenoid metabolites with significant differences from D003 and D009 kernel during three developmental stages (18, 25, and 32 DAP). **(A)** Total content of carotenoids. **(B)** Total content of carotenes. **(C)** Total content of xanthophylls. **(D)** Carotene metabolites with significant differences. **(E)** Xanthophyll metabolites with significant differences. Data are presented as mean ± SD from three biological replicates (*n* = 3). ^**^represents a significant difference at *P* < 0.01 using Student’s *t*-test; the exact *P*-values for the carotenoid metabolites are provided in **Supplementary Table S3**.

To explore the correlation between carotenoid content and maize kernel hardness, kernels from different developmental stages (18, 25, and 32 DAP) of D003 and D009 were collected for widely targeted metabolomics analysis. Three biological replicates were used for each sample, resulting in 18 metabolomics data points. Principal component analysis (PCA) showed a marked similarity among the three biological replicates within each treatment, indicating the reliability and reproducibility of the data (**Figure 4A**). In total, 68 carotenoid metabolites were identified, consisting of 7 carotenes and 61 xanthophylls (**Supplementary Table S2**). The hierarchical cluster heatmap showed that there were 38 carotenoid metabolites with differences between D003 and D009 (**Supplementary Figure S1**). Further analysis revealed that the total carotenoid content of D003 was significantly higher than that of D009 (**Figure 5A**). In the subclass of carotenoids, the carotene and xanthophyll contents of D003 were also significantly higher than those of D009 (**Figures 5B, C**). Among the metabolites of carotene, (E/Z)-phytoene, α-carotene, β-carotene, and lycopene of D003 were significantly higher than those of D009 (**Figure 5D**). Among the metabolites of xanthophyll, α-cryptoxanthin, β-cryptoxanthin, zeaxanthin, zeaxanthin palmitate, violaxanthin-myristate-laurate, rubixanthin palmitate, and lutein of D003 were significantly higher than those of D009 (**Figure 5E**). We constructed a Venn diagram of the three comparative groups, revealing that there were 20 differential accumulation metabolites (DAMs) that were specifically expressed during the three developmental stages in the kernel (**Figure 4B**). The Kyoto Encyclopedia of Genes and Genomes (KEGG) enrichment analysis indicated that these 20 DAMs were significantly enriched in five KEGG pathways (**Figures 4C–E**). These results showed that higher carotenoid content, especially carotene and xanthophyll, increased endosperm hardness.

### 3.4 Transcriptomics analysis found that starch and sucrose metabolism were important regulated pathways

**Figure 6:**
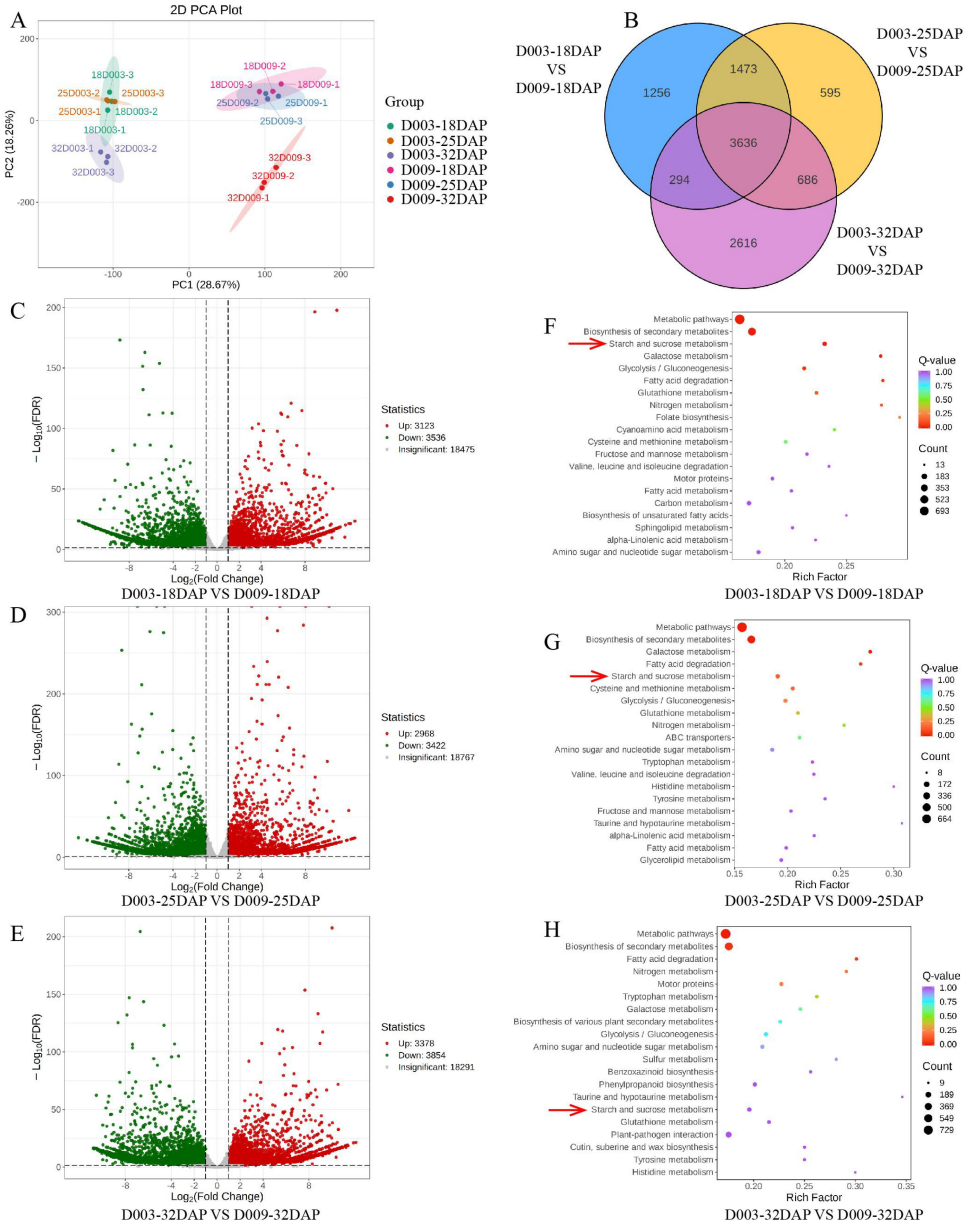
Transcriptomic analysis of D003 and D009. **(A)** PCA of transcriptome data derived from D003 and D009 kernels during three developmental stages (18, 25, and 32 DAP). **(B)** Venn diagram of DEGs. **(C–E)** Volcano plot of DEGs from D003 and D009 at 18, 25, and 32 DAP. **(F–H)** KEGG enrichment analysis of DEGs at 18, 25, and 32 DAP. The color of the circle denotes the *P*-value, and the size of the circle denotes the number of DEGs in the pathway.

To explore the potential molecular regulatory network for kernel hardness formation in maize, kernels from different developmental stages (18, 25, and 32 DAP) of D003 and D009 were also collected for RNA-seq. Three biological replicates were used for each sample, resulting in 18 transcriptomic data points. PCA showed a marked similarity among the three biological replicates within each treatment (**Figure 6A**). In line with the PCA results, correlation analysis showed a marked similarity among the three biological replicates within each treatment, indicating the reliability and reproducibility of the data (**Supplementary Figure S2A**). Then, differentially expressed genes (DEGs) were analyzed in D003 and D009 to gain further insights into the transcriptional changes. In the 18 DAP comparison group, a total of 6,659 DEGs were identified, of which 3,123 were upregulated and 3,536 were downregulated (**Figure 6C**). In the 25 DAP comparison group, a total of 6,390 DEGs were identified, of which 2,968 were upregulated and 3,422 were downregulated (**Figure 6D**). In the 32 DAP comparison group, a total of 7,232 DEGs were identified, of which 3,378 were upregulated and 3,854 were downregulated (**Figure 6E**). There were a total of 3,636 shared DEGs in the three comparative groups (**Figure 6B**). Then, we performed the KEGG enrichment analysis on the 3,636 DEGs to identify the main regulated pathways (**Figures 6F–H**; **Supplementary Figure S2B**). The results revealed that the “metabolic pathways” was the vital pathway generated by KEGG enrichment analysis. In the biosynthesis of secondary metabolite pathways, the “starch and sucrose metabolism” were highly enriched in the D003-18DAP vs. D009-18DAP, D003-25DAP vs. D009-25DAP, and D003-32DAP vs. D009-32DAP comparison groups.

### 3.5 qRT-PCR validation of key genes in starch and sucrose metabolism pathways

**Figure 7:**
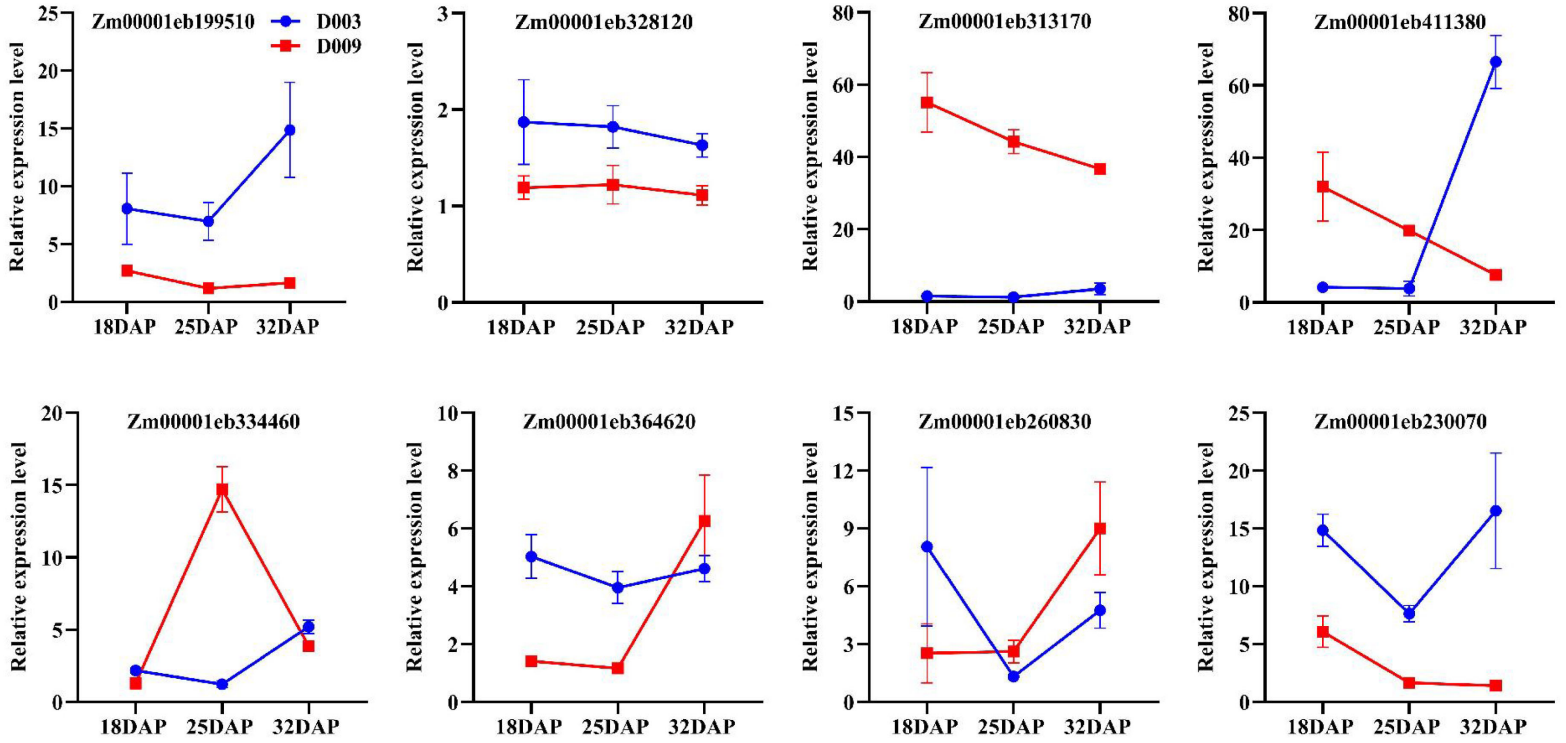
Expression profiles of eight candidate genes by qRT-PCR. Data are presented as mean ± SD from three biological replicates (*n* = 3). The error bars indicate standard errors.

To experimentally validate the RNA-seq results implicating starch and sucrose metabolism in endosperm hardness, we performed qRT-PCR on eight candidate genes identified from this pathway (**Supplementary Figures S3–S6**). These genes—*Zm00001eb199510*, *Zm00001eb328120*, *Zm00001eb313170*, *Zm00001eb411380*, *Zm00001eb334460*, *Zm00001eb364620*, *Zm00001eb260830*, and *Zm00001eb230070*—were selected based on their significant differential expression across multiple developmental stages (18, 25, and 32 DAP) in D003 vs. D009. The results confirmed a strong concordance between RNA-seq and qRT-PCR data, and all eight genes exhibited expression trends consistent with the transcriptome profiles (**Figure 7**). Notably, *Zm00001eb313170* (sucrose synthase) was significantly downregulated in D003 at all stages, suggesting that starch synthesis may be blocked and affect the hardness of the kernel endosperm. These results robustly corroborate the RNA-seq findings and underscore the pivotal role of starch and sucrose metabolism genes, especially the candidate gene *Zm00001eb313170*, in modulating endosperm hardness through regulation of starch granule formation.

## 4 Discussion

Maize kernel hardness, primarily determined by the structural and compositional features of the endosperm, is a vital agronomic trait affecting processing quality, nutritional value, and postharvest performance. While the morphological distinctions between vitreous and starchy endosperms are well-documented (Gibbon and Larkins, 2005; Wang et al., 2020), the metabolic and transcriptional regulatory networks governing these differences remain insufficiently characterized. In this study, we applied an integrated multi-omics approach to dissect the molecular and metabolic basis of kernel hardness in two contrasting maize inbred lines (D003, vitreous; D009, starchy) across key developmental stages. Our findings reveal a coordinated regulation of starch and sucrose metabolism and carotenoid accumulation, providing novel insights beyond previous studies that often focused on single pathways or static developmental stages.

Cytological analyses provided essential insights into the structural foundations of kernel hardness. Both inbred lines began starch accumulation approximately 18 DAP, but D003 exhibited a more rapid and pronounced increase in starch grain size and packing density, culminating in a tightly organized cellular architecture by 32 DAP. In contrast, D009 showed less progression toward compactness, suggesting limitations in processes related to starch granule filling, shape refinement, and protein matrix organization. D003 endosperms displayed small, densely packed cells containing polygonal starch granules, whereas D009 exhibited larger, irregularly arranged cells filled with loosely packed, predominantly spherical granules. These structural differences correspond with established associations between vitreousness and the presence of tightly packed starch granules embedded in a continuous protein matrix, which restricts light scattering and fracture propagation, thereby enhancing hardness and mechanical resilience (Wang et al., 2012; Clore et al., 1996; Wang et al., 2024).

Metabolomic profiling revealed a consistent and pronounced difference in carotenoid accumulation between the two lines. D003 kernels exhibited significantly higher total carotenoid content across all developmental stages, encompassing both carotenes [(E/Z)-phytoene, α-carotene, β-carotene, lycopene] and xanthophylls (α-cryptoxanthin, β-cryptoxanthin, zeaxanthin, zeaxanthin palmitate, violaxanthin-myristate-laurate, rubixanthin palmitate, lutein). The KEGG pathway enrichment analysis of DAMs identified carotenoid biosynthesis as significantly enriched in association with the vitreous phenotype. Although carotenoids are widely recognized for their nutritional and pigmentary roles, their involvement in modulating endosperm texture has received comparatively little attention. Previous studies have suggested such links—for example, *Ven1* influences β-carotene levels and affects starch granule membrane integrity and protein–starch interactions (Wang et al., 2020), while zeaxanthin tends to be enriched in vitreous endosperm (Egesel et al., 2003). Our findings strongly reinforce the positive correlation between carotenoid accumulation—especially specific carotenes and xanthophylls—and kernel hardness. We hypothesize that carotenoids, owing to their lipophilic nature, may integrate into plastid membranes, protein bodies, and starch granule surfaces, potentially modulating membrane fluidity and stability as well as interactions between the protein matrix and starch granules, thereby contributing to the rigidity and dense architecture characteristic of vitreous endosperm (Howitt and Pogson, 2006; Koul et al., 2016).

Transcriptomic analysis added a crucial dimension by revealing transcriptional regulation of key metabolic pathways associated with kernel hardness. The most prominent finding was the consistent enrichment of the starch and sucrose metabolism pathway among the DEGs shared across all stages and specific pairwise comparisons. This pathway is central to kernel development, providing the carbon skeletons and energy required for endosperm filling, while also contributing sugar-derived signaling molecules (Huang et al., 2021; Sekhon et al., 2011). Eight candidate genes from this pathway were selected for qRT-PCR validation, with particular attention to a sucrose synthase gene (*Zm00001eb313170*), which was consistently downregulated in D003 across all stages. Sucrose synthase (Susy) plays a pivotal role in cleaving sucrose into UDP-glucose and fructose, key substrates for starch biosynthesis and other metabolic routes (Carlson et al., 2002; Li et al., 2013; Deng et al., 2020). Reduced expression of *Susy* in D003 may indicate several possibilities: 1) a reallocation of sucrose flux toward alternative metabolic or storage sinks; or 2) a slower sucrose turnover rate, which may alter sugar signaling and, in turn, affect cellular growth, starch granule morphogenesis, and storage compound deposition. This phenomenon is associated with altered carbon flux and sugar signaling, which may influence starch granule morphology and packing density. However, the causal roles of these genes require functional validation.

Integrating these findings, we propose a model in which starch and sucrose metabolism serve as the principal drivers of endosperm structure and hardness, modulated at the transcriptional level. Enzymes such as sucrose synthase influence carbon allocation and sugar signaling, affecting the biosynthesis, morphology (e.g., polygonal shape), and packing of starch granules. Simultaneously, upregulation of carotenoid biosynthesis contributes synergistically by reinforcing internal membrane systems and structural interfaces within the developing endosperm (e.g., starch granule surfaces, protein matrices), thereby supporting the compact organization established by carbohydrate metabolic regulation. This dual mechanism highlights the convergence of primary (starch and sucrose) and specialized (carotenoid) metabolic pathways in determining the physical attributes of maize kernels. Our integrated analysis thus extends existing models by elucidating how transcriptional and metabolic modules interact dynamically across development to shape endosperm hardness. The potential co-regulation of these pathways at the transcriptional level merits further exploration. Despite the significant insights provided by this study, some limitations must be acknowledged. Functional validation of the candidate genes identified—particularly *Zm00001eb313170* and others involved in starch and sucrose metabolism—using reverse genetics approaches (e.g., knockout mutants, overexpression lines, and complementation studies) is essential to establish causality in determining endosperm hardness.

## 5 Conclusion

In conclusion, our integrated metabolomic and transcriptomic investigation reveals that maize kernel hardness is jointly regulated by transcriptional modulation of starch and sucrose metabolism and by elevated carotenoid accumulation at the metabolic level. The vitreous phenotype observed in D003 results from a developmental program characterized by tightly packed endosperm cells with polygonal starch granules embedded in a robust matrix. This phenotype is driven by distinct transcriptional regulation of carbohydrate metabolism genes (notably reduced *Susy* expression) and reinforced by the increased presence of specific carotenoids. These findings provide promising molecular targets for breeding and biotechnological strategies aimed at optimizing maize kernel quality. Manipulating the expression of key genes in starch and sucrose metabolism or enhancing carotenoid biosynthetic flux may offer effective means of tailoring kernel hardness to suit specific industrial or nutritional needs. Our study not only advances the mechanistic understanding of kernel hardness but also highlights the power of integrated omics approaches in dissecting complex agronomic traits.

## Data Availability

The original contributions presented in the study are included in the article/**Supplementary Material**. Further inquiries can be directed to the corresponding authors.
