# Supplementary material for: Integrated metabolomic and transcriptomic analyses reveal that starch and sucrose metabolism regulate maize kernel hardness
Source: Front Plant Sci. 2025 Nov 24;16:1688375. doi: 10.3389/fpls.2025.1688375 (PMC12682897; doi:10.3389/fpls.2025.1688375)
Supplement: Supplementary file 1 [file DataSheet1.doc]

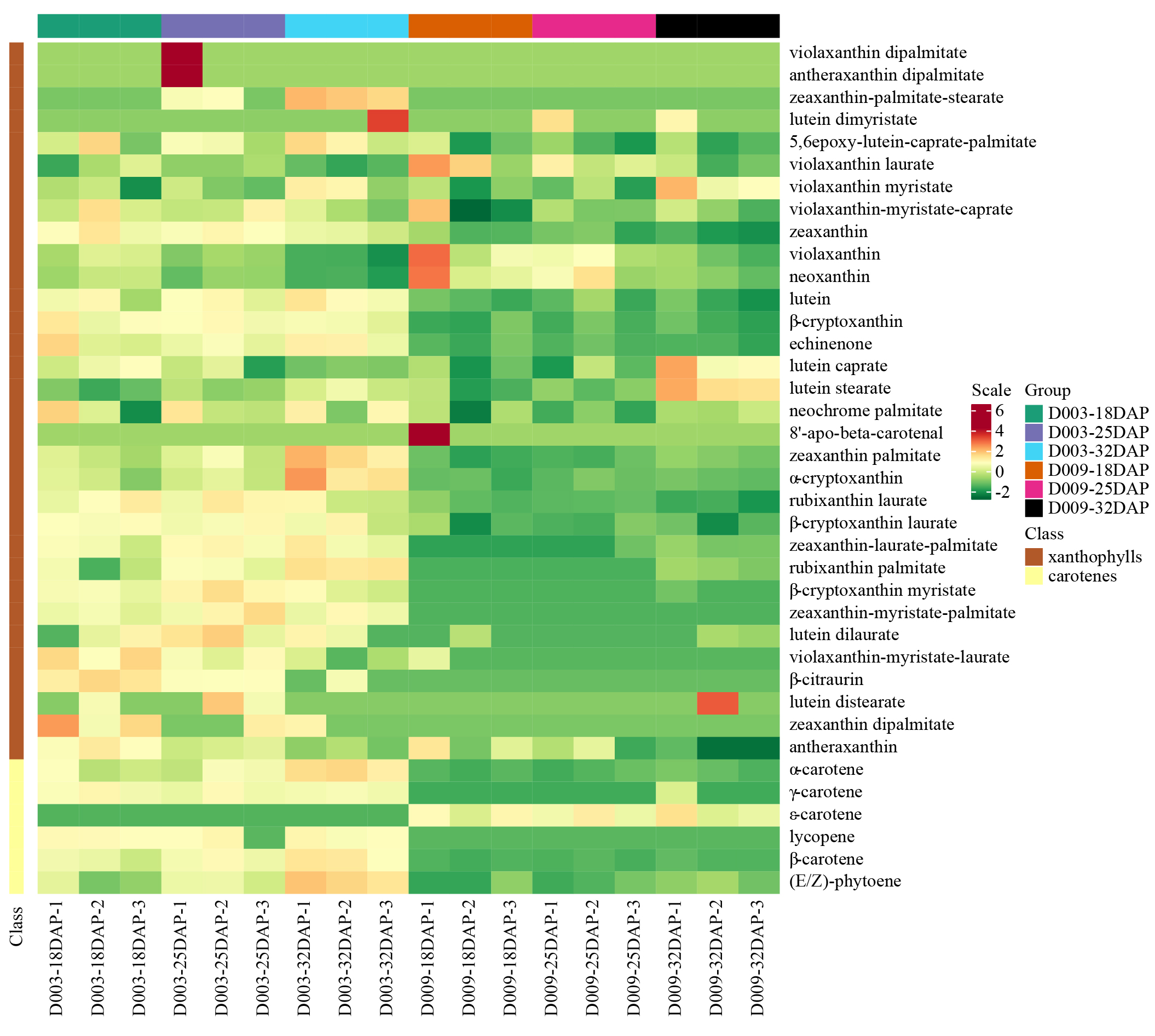


Supplementary Figure S1. Hierarchically clustered heatmap of the differential 38 carotenoids metabolites from D003 and D009 kernel during three developmental stages (18, 25, 32 DAP).


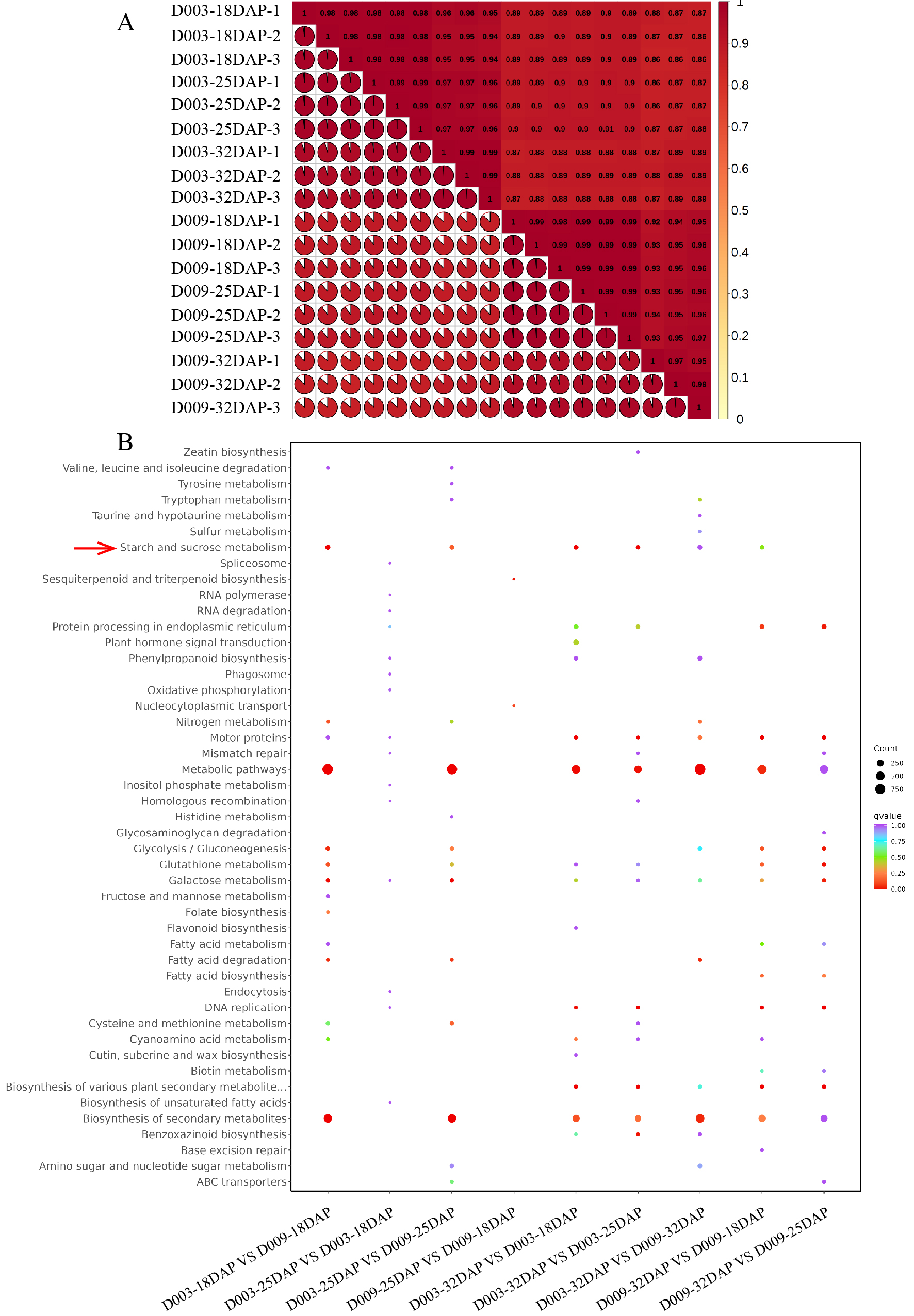


Supplementary Figure S2. Transcriptomic analysis of D003 and D009. (A) Hierarchically clustered heatmap of the gene transcripts. The completeness of the pie chart corresponds to the magnitude of the correlation value. The false color scale is depicted on the right side of the image. (B) Multiple-combination KEGG enrichment analysis of DAMs. Circle color denotes the P-value, circle size denotes the number of DEGs in the pathway.


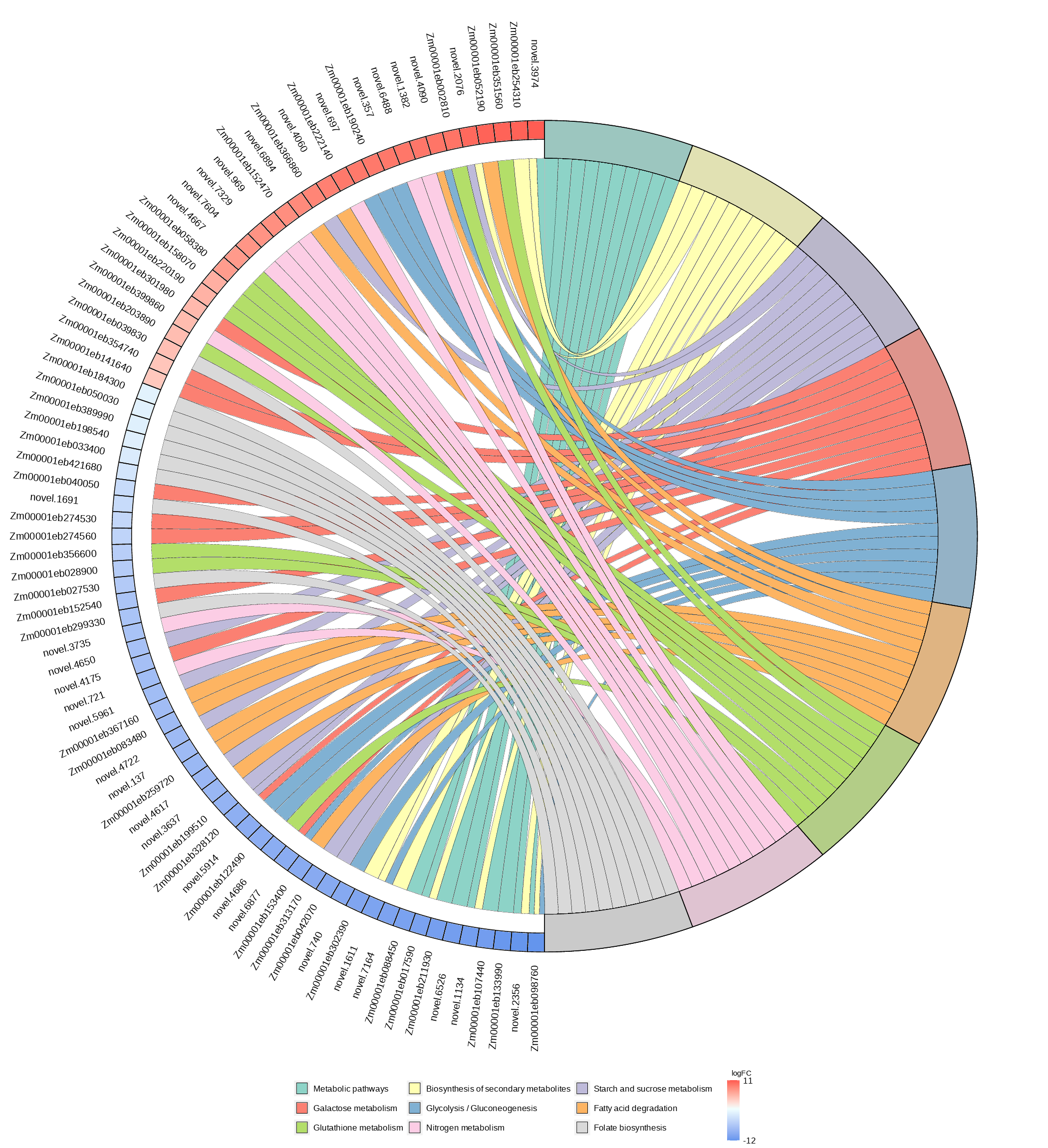


Supplementary Figure S3. The KEGG enrichment chord diagram of DEGs from D003-18DAP VS D009-18DAP.


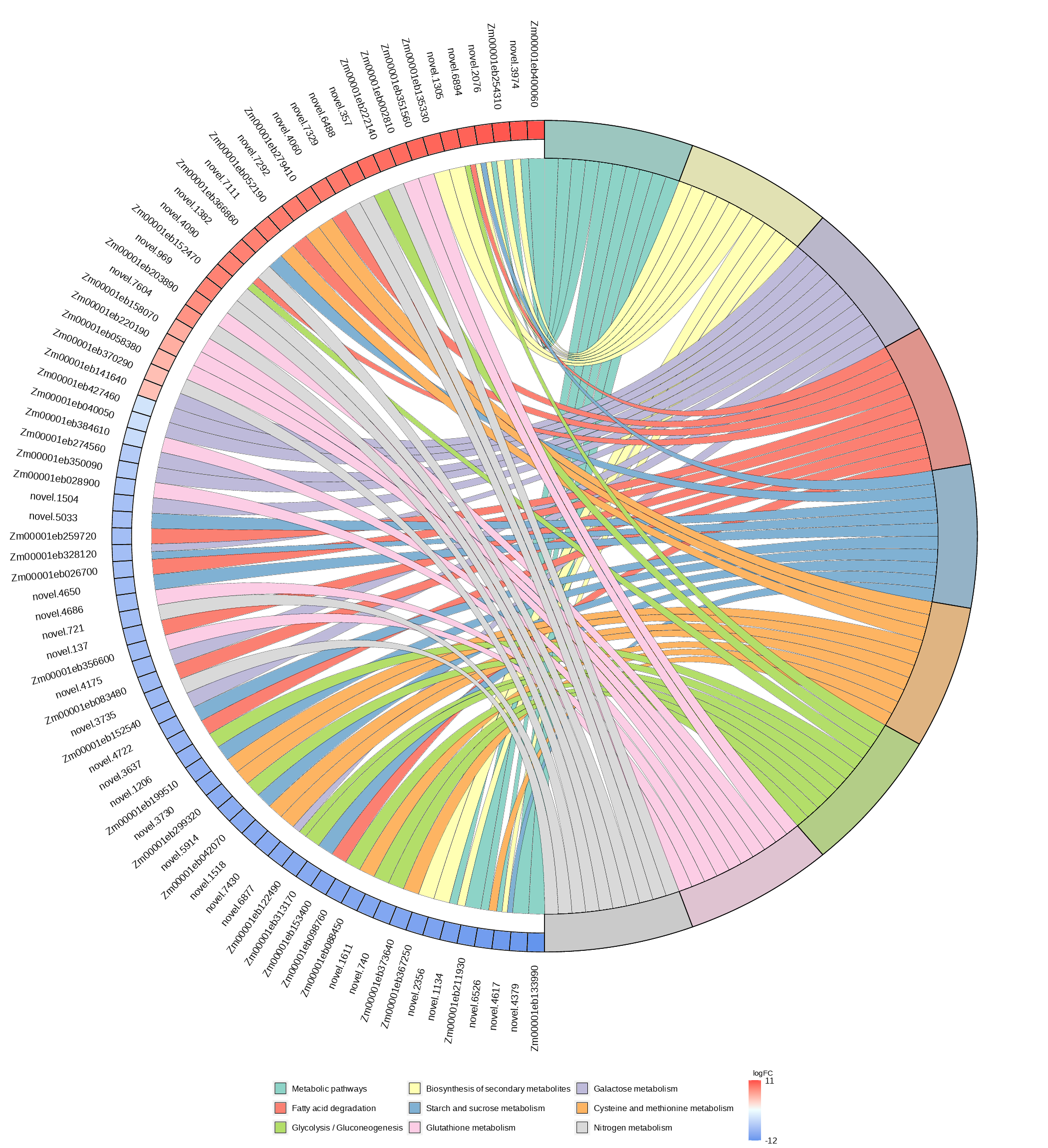


Supplementary Figure S4. The KEGG enrichment chord diagram of DEGs from D003-25DAP VS D009-25DAP.


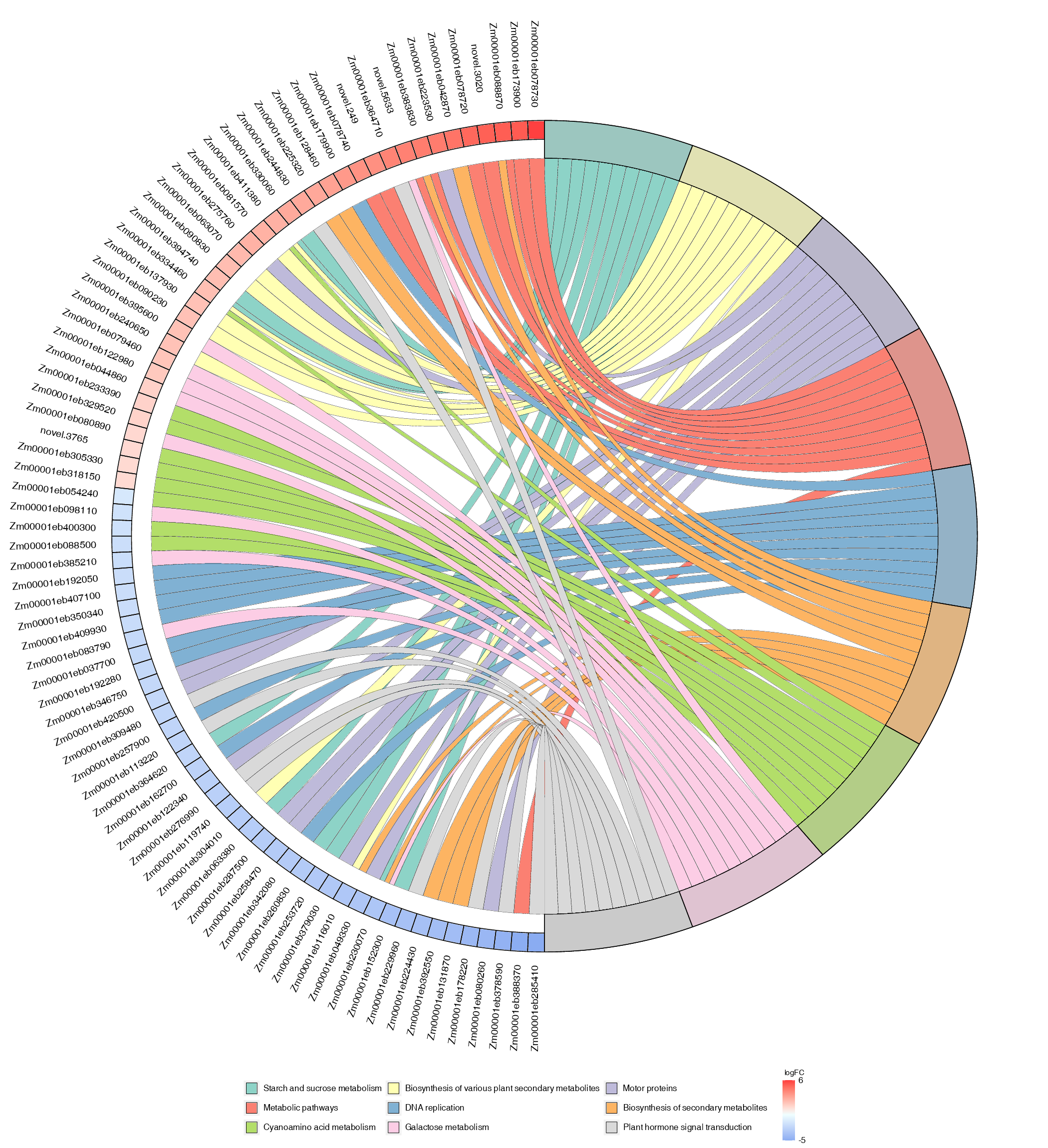


Supplementary Figure S5. The KEGG enrichment chord diagram of DEGs from D003-32DAP VS D003-18DAP.


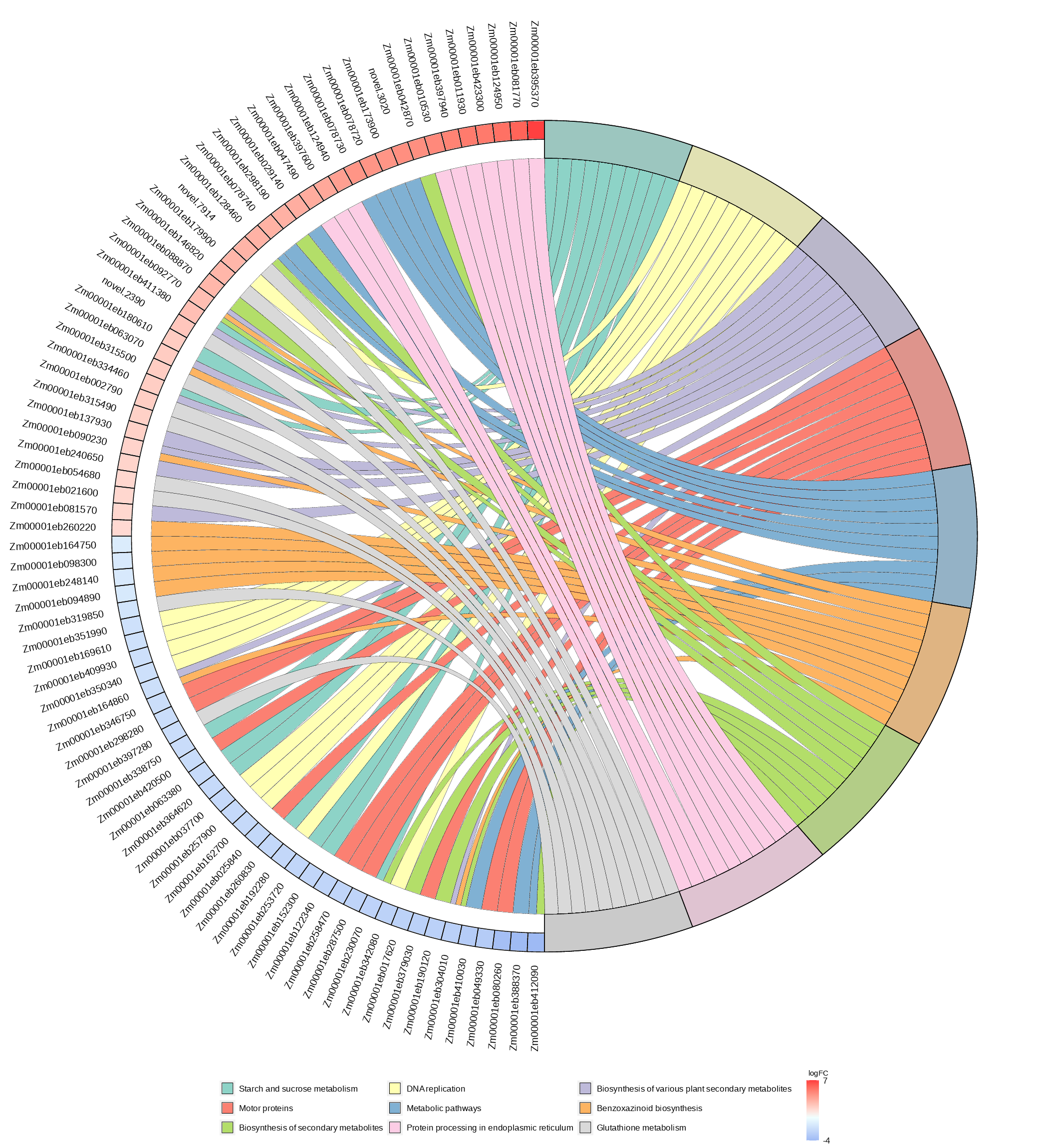


Supplementary Figure S6. The KEGG enrichment chord diagram of DEGs from D003-32DAP VS D003-25DAP.
